# *Turbina oblongata* Protects Against Oxidative Cardiotoxicity by Suppressing Lipid Dysmetabolism and Modulating Cardiometabolic Activities Linked to Cardiac Dysfunctions

**DOI:** 10.3389/fphar.2021.610835

**Published:** 2021-05-20

**Authors:** Ochuko L. Erukainure, Chika I. Chukwuma, Motlalepula G. Matsabisa, Mirranda T. Javu, Veronica F. Salau, Neil A. Koorbanally, Md. Shahidul Islam

**Affiliations:** ^1^ Department of Pharmacology, Faculty of Health Sciences, School of Clinical Medicine, University of the Free State, Bloemfontein, South Africa; ^2^ Center for Quality of Health and Living, Faculty of Health and Environmental Sciences, Central University of Technology, Bloemfontein, South Africa; ^3^ Department of Biochemistry, School of Life Sciences, University of KwaZulu-Natal, Durban, South Africa; ^4^ School of Chemistry and Physics, University of KwaZulu-Natal, Durban, South Africa

**Keywords:** cardiotoxicity, lipid dysmetabolism, metabolomics, turbina oblongata, cardiometabolism

## Abstract

Cardiotoxicity leading to cardiovascular dysfunction and ultimately cardiac failure remains a major global health issue irrespective of race, age and country. Several factors including lipotoxicity, oxidative imbalance, exacerbated angiotensin-converting enzyme (ACE) activity and altered bioenergetics have been implicated in the pathophysiology of cardiovascular diseases. *Turbina oblongata* (E. Mey. ex Choisy) A. Meeuse is among the medicinal plants commonly used traditionally in the treatment and management of various ailments including cardiovascular dysfunctions in South Africa. In the present study, *T. oblongata* was investigated for its cardioprotective mechanism on oxidative-mediated cardiotoxicity by determining its effect on redox imbalance, purinergic and cholinergic dysfunction, and ACE activity as well as lipid dysmetabolism and pathways in iron-induced oxidative cardiac injury. Oxidative injury was induced *ex vivo* in freshly isolated heart by incubating with 0.1 mM FeSO_4_. Treatment was done by co-incubating with *T. oblongata* extract or gallic acid which served as the standard antioxidant. Induction of oxidative cardiac injury led to significant depleted levels of glutathione, triglyceride, HDL-cholesterol, superoxide, catalase and ENTPDase activities, with concomitant elevated levels of malondialdehyde, cholesterol, LDL-cholesterol, ACE, acetylcholinesterase, ATPase and lipase activities. These levels and activities were significantly reversed following treatment with *T. oblongata*. Induction of oxidative injury also caused alterations in lipid metabolites, with concomitant activation of beta oxidation of very long chain fatty acids, plasmalogen synthesis and mitochondrial beta-oxidation of long chain saturated fatty acids pathways. Some of the altered metabolites were restored following treatment with *T. oblongata*, with concomitant inactivation of beta oxidation of very long chain fatty acid pathway. These results indicate the cardioprotective effect of *T. oblongata* against oxidative-mediated cardiotoxicity. This is evidenced by its ability to mitigate lipotoxicity and modulate dysregulated cardiometabolic activities as portrayed by its antioxidative activity and suppressive effects on ACE, acetylcholinesterase and lipase activities, while modulating cardiac lipid dysmetabolism.

## Introduction

Cardiovascular dysfunctions leading to cardiovascular failure has been linked to global early mortalities, with oxidative stress arising from disordered cardiac bioenergetics playing a triggering role (Poulter, 2003; Wold et al., 2005; Li et al., 2019). This is often depicted by cardiac lipid dysmetabolism which is characterized by hyperlipidemia as well as defects in beta-oxidation pathway and mitochondrial oxidative phosphorylation (Antozzi and Zeviani, 1997). Fatty acids (FAs) have been reported as a major energy source for the heart, as they are estimated to meet over 70% of the heart’s energy needs (Drosatos and Schulze, 2013). Alteration in cardiac FAs and their metabolism leading to lipotoxicity has been implicated among the pathomechanism of cardiovascular dysfunctions (Erukainure et al., 2020b; Salau et al., 2020b). Exacerbated activities of cardiac lipase and angiotensin-converting enzyme (ACE), as well as altered cardiac supply of triglycerides from the liver and accumulation of cholesterol in the heart are major contributing factors of lipotoxicity. Lipotoxicity has been linked to increased production and accumulation of free radicals leading to oxidative stress, when the latter overwhelms the cardiac endogenous antioxidant system (Jiménez-González et al., 2020). This crosstalk between lipotoxicity and oxidative stress presents pathogenic mechanisms and progression of cardiotoxicity and other cardiovascular dysfunctions, ultimately leading to heart failure.

*Turbina oblongata* is among the medicinal plants commonly used traditionally in the treatment and management of various ailments in South Africa. It is a perennial herb belonging to the Convolvulaceae family and widely distributed in Botswana, Swaziland, Malawi, Tanzania, Zimbabwe and South Africa (Srivastava and Rauniyar, 2020). In South Africa, the plant is commonly found in all provinces except the Western Cape and it is locally referred to as *ubhoqo* (Semenya et al., 2018; Srivastava and Rauniyar, 2020). Its other synonyms are *Ipomoea atherstonei*, *Ipomoea lambtoniana*, *Ipomoea oblongata* and *Ipomoea randii* (Foden and Potter, 2005). The plant leaves have been reported for their wound healing properties and anticancer activities (Hutchings et al., 1996; Kose et al., 2015). The plant is also traditionally used in the treatment of diarrhea, respiratory infections, hypertension, impotency, renal disease, and sexually transmitted infections (Srivastava and Rauniyar, 2020). These properties can be attributed to its phytochemical constituents which have been reported to consist of flavonoids, terpenoids, glycosides, steroids, anthocyanins, and alkaloids.

The present study investigated the cardioprotective mechanism of *T. oblongata* on oxidative-mediated cardiotoxicity by reporting its effect on redox imbalance, purinergic and cholinergic dysfunction, ACE activity and lipid dysmetabolism in iron-induced oxidative cardiac injury.

## Materials and Methods

### Plant Material and Extraction

*Turbina oblongata* was collected from Eastern Cape Province, South Africa (GPS coordinates: 32°44′20.1″S 26°54′58.3″E). The plant sample was identified and authenticated at the Geo Potts herbarium (BLFU), University of the Free State (specimen voucher number: BLFU MGM0019).

The plant sample was air-dried after washing and was blended to fine powder. The powdered sample (100 g in a 1:5 mass:solvent ratio in ml) was subjected to sequential extraction using solvents of increasing polarity vis-à-vis hexane, dichloromethane (DCM), methanol and dichloromethane (MeOH:DCM; 1:1, v/v) and methanol (MeOH). Each extraction was carried out at ambient room temperature for 24 h. After extraction, each extract was filtered and subsequently concentrated *in vacuo* with an R–215 rotary evaporator (Buchi, Switzerland). Concentrated samples were collected into glass vials and stored at 4 C.

### Enzyme Inhibitory Activity

The concentrated extract samples were investigated for their anti-hypertensive activities by determining their ability to inhibit the activity of ACE *in vitro* (Shalaby et al., 2006). Based on the ACE inhibitory activities, the DCM extract was selected for further evaluation for its anti-hypertensive potential by determining its ability to inhibit the activity of renin using modified standard method (Udenigwe et al., 2009). The DCM extract, based on its activity on ACE and Renin, was chosen for further *ex vivo* studies.

### Induction of Oxidative Cardiac Injury *Ex Vivo*

Hearts harvested from Sprague Dawley rats were homogenized in 50 mM sodium phosphate buffer (pH 7.5; with 1% Triton X-100). The homogenates were then centrifuged at 15,000 rpm at 4°C for 10 min to obtain the tissue supernatants.

A 100 µl of the cardiac tissue supernatant was incubated with 0.1 mM FeSO_4_ (pro-oxidant) and different concentrations (30, 60, 120 and 240 μg/ml) of the DCM extract of *T. oblongata* at 37°C for 30 min according to previously published methods (Erukainure et al., 2020a; Salau et al., 2020b). Reaction samples without the extract served as the negative control (untreated), while reaction sample without the pro-oxidant and extract served as the normal control. Gallic acid was used as the standard antioxidative drug.

The study was conducted under the approved guidelines of the Animal Ethics Committee of the University of KwaZulu-Natal, Durban, South Africa (Protocol approval number: AREC/020/017D).

### Determination of Oxidative Stress Parameters

#### Reduced Glutathione Level

This was determined according to Ellman’s method (Ellman, 1959). Briefly, 100 μl of the reaction samples was deproteinized with 300 μl of 10% TCA and centrifuged at 3,500 rpm for 5 min. 200 μl of the mixture was added to 50 μl of Ellman’s reagent in a 96-well plate and incubated for 5 min. Absorbance was measured at 415 nm and GSH level was extrapolated from a GSH standard curve.

#### Superoxide Dismutase Enzyme Activity

This was determined with slight modification of a previously established method (Kakkar et al., 1984). Briefly, 15 μl of the reaction samples was mixed with 170 μl of 0.1 mM diethylenetriaminepentaacetic acid (DETAPAC) in a 96-well plate. Then 15 μl of 1.6 mM 6-hydroxydopamine (6-HD) was added to the mixture and swirled. Absorbance was immediately read at 492 nm for 3 min at 1 min interval.

#### Catalase Activity

Catalase activity was determined using a previously established protocol (Aebi, 1984). Briefly, 100 μl of the reaction samples was mixed with 340 μl of 50 mM phosphate buffer (pH 7.0). Thereafter, 150 μl of 2 M hydrogen peroxide was added to the mixture and incubated for 5 min. Absorbance was measured at 240 nm at 1 min interval for 3 min.

#### Lipid Peroxidation

Lipid peroxidation was carried out by measuring the malondialdehyde (MDA) level of the reaction samples using a previously established method (Chowdhury and Soulsby, 2002). In brief, a reaction mixture made up of equal volumes (100 μl) of the reaction samples and 8.1% SDS solution, 1 ml of 0.25% thiobarbituric acid (TBA) and 375 μl of 20% acetic acid was boiled for 1 h. Absorbance was measured at 532 nm and MDA levels were extrapolated from a MDA standard curve.

### Determination of ACE Activity

ACE activity was determined spectrophotometrically by a slight modification of Holmquist’s method (Holmquist et al., 1979), using N-[3-(2-Furyl) acryloyl]-L-phenylalanyl-glycyl-glycine (FAPGG) as substrate. Briefly, 200 μl of the reaction samples were incubated with 1 ml of 0.5 mM FAPGG for 10 min at 37°C. Absorbance was measured at 345 nm at 2 min intervals. ACE activity was measured as the rate of reaction (ΔA/min) as expressed below:

$$\mathrm{ACE}\;\mathrm{activity}=\frac{\left(\mathrm{AI-AF}\right)\mathrm{Sample}-\left(\mathrm{AI-AF}\right)\mathrm{Blank}}{\mathrm{Time interval}\;\left(\min\right)}\;$$

Where AI = initial absorbance; AF = final absorbance.

### Determination of Cholinergic Enzymes Activity

Cholinergic enzyme activity was determined by analyzing the acetylcholinesterase activity of the reaction samples using the Ellman’s procedure (Ellman et al., 1961). A reaction containing 20 μl of the reaction samples, 10 μl of Ellman’s reagent (3.3 mM, pH 7.0) and 50 μl of phosphate buffer (0.1 M, pH 8) was incubated for 20 min at 25°C. Then 10 μl of 0.05 M acetylcholine iodide was added to the reaction samples and absorbance was measured at 412 nm at 3 min intervals.

### Determination of Purinergic Enzymes Activities

The purinergic enzyme activities of the reaction samples were determined by analyzing the activities of adenylpyrophosphatase (ATPase) (Adewoye et al., 2000; Erukainure et al., 2017) and ecto-nucleoside triphosphate diphosphohydroase (ENTPDase) (Akomolafe et al., 2017) respectively.

#### ATPase Activity

Briefly, a reaction mixture containing 100 μl of the reaction samples, 100 μl of 5 mM KCl, 650 μl of 0.1 M Tris-HCl buffer, and 20 μl of 50 mM ATP was incubated in a shaker for 30 min at 37°C. Then 1.25% ammonium molybdate and 500 μl of a distilled water were added to terminate the reaction. Thereafter, a 500 μl of a freshly prepared 9% ascorbic acid was added to the mixture and allowed to stand for 30 min. Absorbance was measured at 660 nm.

#### ENTPDase Activity

Briefly, 20 μl of the reaction samples was incubated with a mixture containing 200 μl of the reaction buffer (1.5 mM CaCl_2_, 5 mM KCl, 0.1 mM EDTA, 10 mM glucose, 225 mM sucrose and 45 mM Tris-HCl) for 10 min at 37°C. 20 μl of 50 mM ATP was then added to the reaction mixture and further incubated in a shaker for 20 min at 37°C. Reaction was halted by adding 200 μl of 10% TCA, followed by 200 μl of 1.25% ammonium molybdate and a freshly prepared 9% ascorbic acid. The mixture was allowed to stand on ice for 10 min. Absorbance was read at 600 nm.

### Determination of Lipase Activity

The lipase activity of the reaction samples was determined by a little modification of a previously established method (Kim et al., 2010). A 100 μl of the reaction samples was incubated with 169 μl of Tris buffer (100 mM Tris–HC1 and 5 mM CaCl_2_, pH 7.0) at 37°C for 15 min 5 μl of 10 mM *p*-NPB (*p*-nitrophenyl butyrate in dimethyl formamide) was added to the mixture and further incubated for 15 min at 37°C. Absorbance was measured at 405 nm at 1 min interval. Lipase activity was expressed as the rate of reaction (ΔA/min).

### Determination of Cardiac Lipid Profile

Cardiac tissue supernatants were incubated overnight (12 h) with 0.1 mM FeSO_4_ and 240 μg/ml of *T. oblongata* DCM extract or gallic acid as described above. The samples were centrifuged at 15,000 rpm for 10 min at 4°C. The supernatants were immediately assayed for lipid profile levels including, total cholesterol, triglycerides, and HDL-cholesterol using an Automated Chemistry Analyzer (Labmax Plenno, Labtest Co. Ltd., Lagoa Santa, Brazil) with commercial assay kits according to manufacturer’s manual.

### Metabolite Extraction and Profiling

As described above, the reaction mixture was prepared and incubated overnight (12 h). Lipid metabolites were then extracted from the samples and subjected to GC-MS metabolic profiling (Ralston-Hooper et al., 2011) with slight modification. Briefly, cold chloroform was mixed with the reaction samples in a ratio of 5:1 and vortexed for 1 min. The mixture was incubated on ice for 20 min and centrifuged at 15,000 rpm for 10 min at 4°C to yield two liquid phases. The lower phase (chloroform) layer containing the lipid metabolites and other non-polar metabolites was collected and subsequently profiled with GC-MS.

### GC-MS Analysis of Lipid Metabolites

The chloroform layer which contains the lipid and other non-polar metabolites were analyzed with GC-MS (Agilent technologies 6,890 series GC coupled with (an Agilent) 5,973 Mass Selective Detector and driven by Agilent Chemstation software). The operating parameters include: Column: HP-5MS capillary column (30 m × 0.25 mm ID, 0.25 μm film thickness, 5% phenylmethylsiloxane); Carrier gas: ultra-pure helium; Flow rate: 1.0 ml min^−1^ and a linear velocity of 37 cm s^−1^; Injector temperature: set at 250°C. Initial oven temperature: 60°C, programmed to 280°C at the rate of 10°C min^−1^. Injection: 1 μl made in split mode at a split ratio of 20:1; Electron ionization mode: 70 eV; Electron multiplier voltage: at 1859 V; Ion source temperature: 230°C; Quadrupole temperature: 150°C; Solvent delay: 4 min; Scan range: 50–70 amu. Lipid metabolites were identified using an inbuilt NIST library.

### Metabolic Pathway Analysis

To identify the relevant pathways involved in lipid metabolism in the protective role of *T. oblongata* on oxidative cardiotoxicity, the identified lipid metabolites were subjected to pathway enrichment analysis using the MetaboAnalyst 4.0 online server (Chong et al., 2018).

### High-Performance Liquid Chromatography

HPLC-diode array detection analysis was performed on the DCM extract using an Agilent 1,100 series (Agilent, Waldbronn, Germany) instrument equipped with photo diode array, autosampler, column thermostat and degasser. A Phenomenex: Luna 5 µm C_18_ (2) (150 × 4.6 mm; 5 μm particle size) column was used as the stationary phase. Water containing 0.1% of formic acid (A) and acetonitrile (B) served as mobile phases at a flow rate of 1 ml/min. Gradient elution was applied as follows: Initial ratio 95% A: 5% B, keeping for 10 min, changed to 90% A: 10% B in 10 min, changed to 70% A: 30% B in 10 min, to 50% A: 50% B in 10 min, maintaining for 0.5 min and back to initial ratio in 0.5 min. Temperature was set to 30°C. Extract or standards were dissolved in HPLC grade methanol (2 mg/ml) and the injection volume was 20.0 μl. Chromatograms were recorded at 254 nm.

### Statistical Analysis

One-way analysis of variance (ANOVA) was used in analyzing the data and presented as mean ± SD. Significant differences between means at *p* < 0.05 were obtained with the Tukey’s HSD-multiple range post-hoc test. Statistical analyses were done using IBM Statistical Package for the Social Sciences (SPSS) for Windows, version 23.0 (IBM Corp., Armonk, NY, United States). The GC-MS identified metabolites were subjected to statistical analysis (distinct changes and distribution) using the MetaboAnalyst 4.0 online server (Chong et al., 2018). Graphs were prepared using Microsoft Excel Spreadsheet and MetaboAnalyst 4.0 online server.

## Results

Except for MEOH:DCM and DCM extracts, the extracts had no inhibitory effect on ACE activity as shown in Supplementary Figure S1A. The MEOH:DCM and DCM extracts significantly (*p* < 0.05) inhibited the enzyme activity in a dose dependent manner. This is further depicted by their IC_50_ values of 167 and 81.1 μg/ml, respectively (Table 1) and compared favourably with that of the standard ACE-inhibitor captopril oil (Supplementary Figure S1B).

**TABLE 1:** IC50 values for ACE and renin inhibitory activities of *T. oblongata* extracts.

Activities	HEX	MEOH	DCM:MEOH (1:1)	DCM	Aqueous	Captopril	Aliskiren
	(µg/ml)						
ACE inhibition	ND	364	167	81.1	ND	1.31	ND
Renin inhibition	ND	ND	ND	127	ND	ND	13.4

HEX, hexane extract; MEOH, methanol extract; DCM:MEOH, dichloromethane and methanol extract; DCM, dichloromethane extract. ND = not determined.

The DCM extract having displayed the best ACE inhibitory activity, and was recommended for further investigated for its renin inhibitory activity. The extract significantly (*p* < 0.05) inhibited the ACE activity dose dependently (Supplementary Figure S2A), with an IC_50_ value of 127 μg/ml (Table 1). This activity compared favorably with that of Aliskiren (Supplementary Figure S2B and Table 1).

**FIGURE 1:**
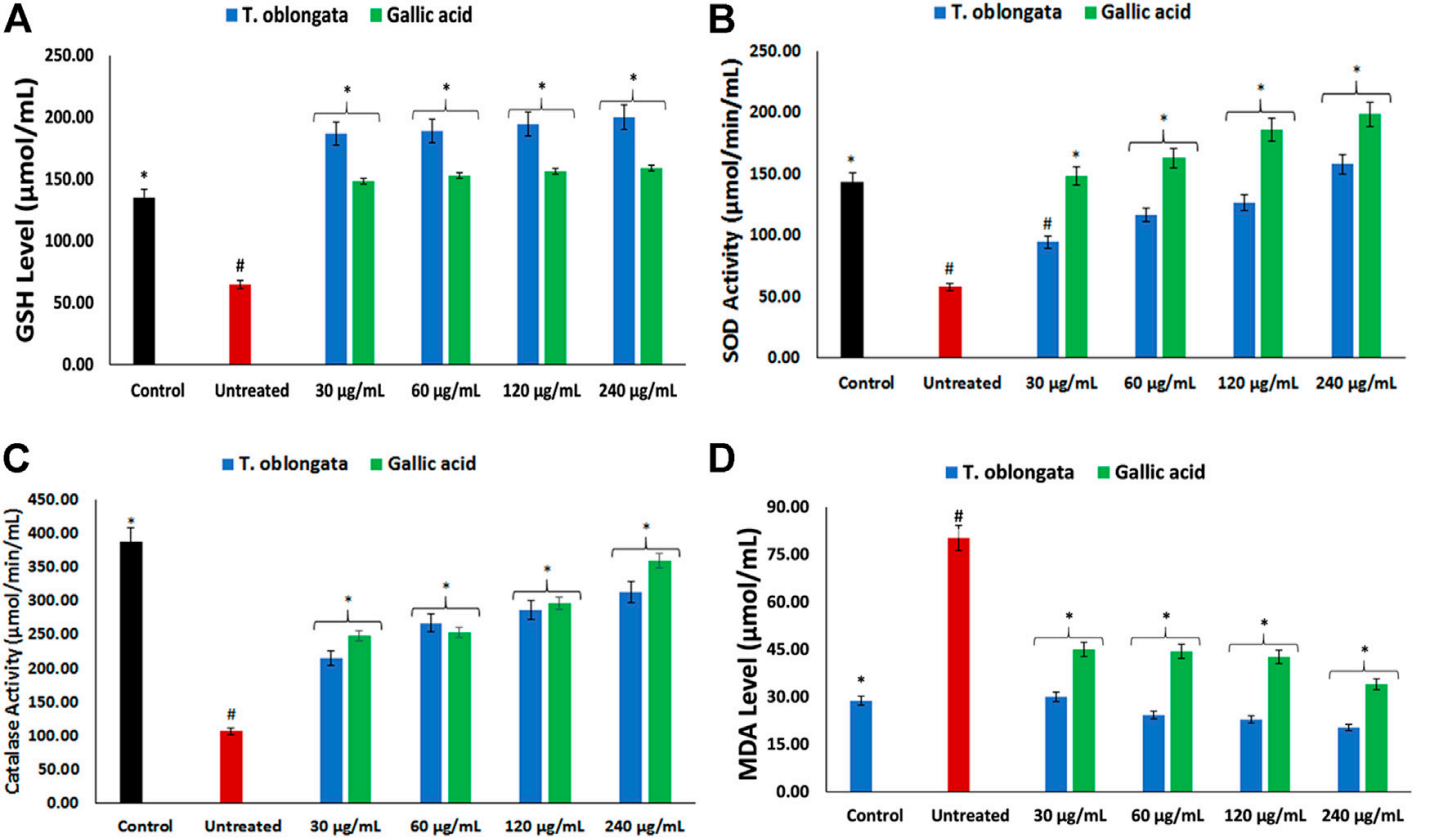
Effect of *T. oblongata* on **(A)** GSH level, **(B)** SOD, **(C)** catalase and **(D)** MDA level in oxidative cardiotoxicity. Data = mean ± SD; *n* = 3. *Statistically significant compared to untreated cardiac tissues; #statistically significant compared to normal cardiac tissues.

Induction of oxidative cardiac injury led to significant (*p* < 0.05) decreased levels of GSH, SOD and catalase activity, with concomitant elevated level of MDA as depicted in Figures 1A–D. Following treatment with the DCM extract, these levels and activities were significantly (*p* < 0.05) reversed to near normal. Except for GSH level, these reversals were dose-dependent and compared favourably with gallic acid.

**FIGURE 2:**
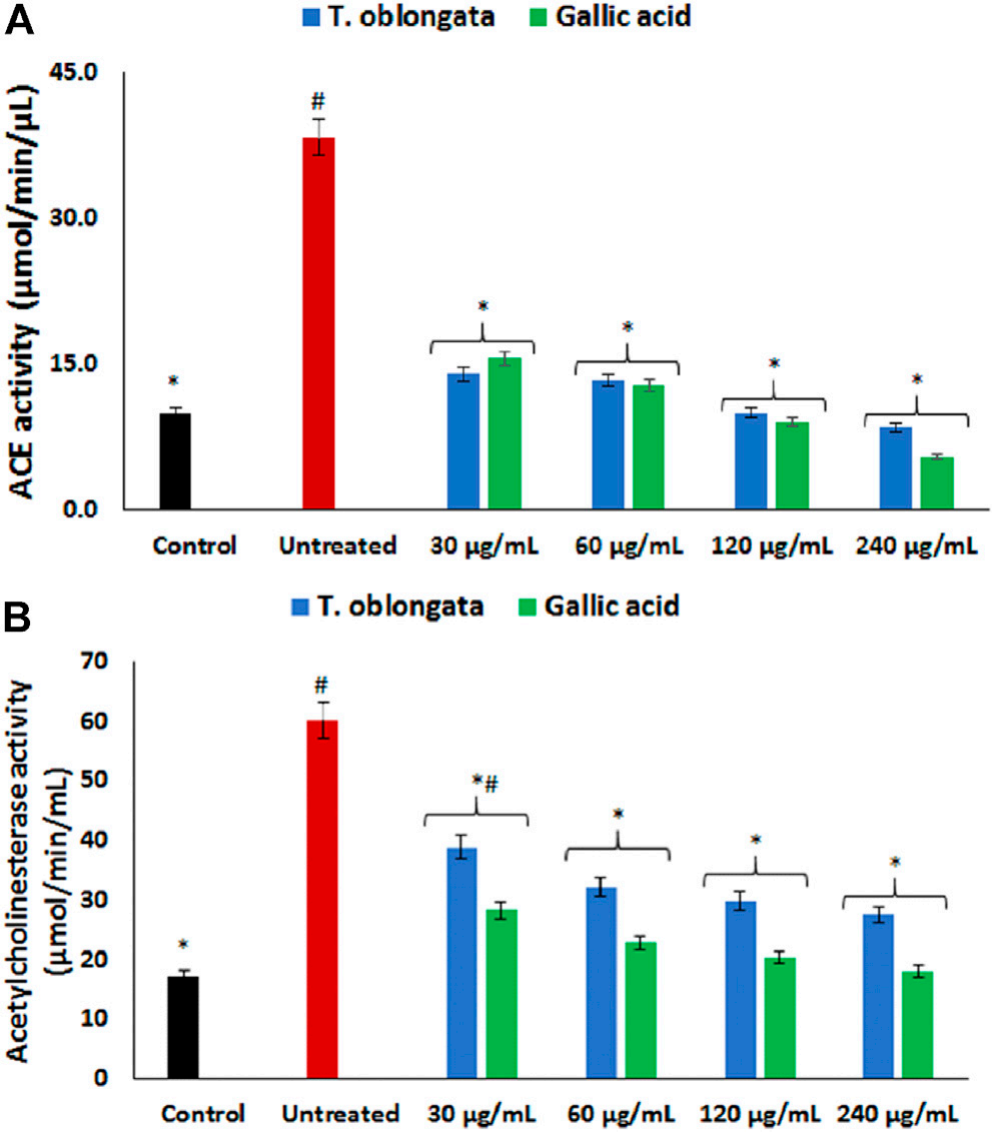
Effect of *T. oblongata* on **(A)** ACE and **(B)** acetylcholinesterase activities in oxidative cardiotoxicity. Data = mean ± SD; *n* = 3. *Statistically significant compared to untreated cardiac tissues; #statistically significant compared to the control (normal cardiac tissue).

ACE activity was significantly (*p* < 0.05) elevated in cardiac tissues following the induction of oxidative cardiac injury as shown in Figure 2A. Treatment with the DCM extract led to significant (*p* < 0.05) depletion in the activity dose-dependently, depicting an inhibitory effect.

As shown in Figure 2B, induction of oxidative cardiac injury led to significant (*p* < 0.05) elevation of cardiac acetylcholinesterase activity. The activity was significantly (*p* < 0.05) reversed dose-dependently to near noel following treatment with the DCM extract. The reversed activity compared favorably with that of gallic acid.

**FIGURE 3:**
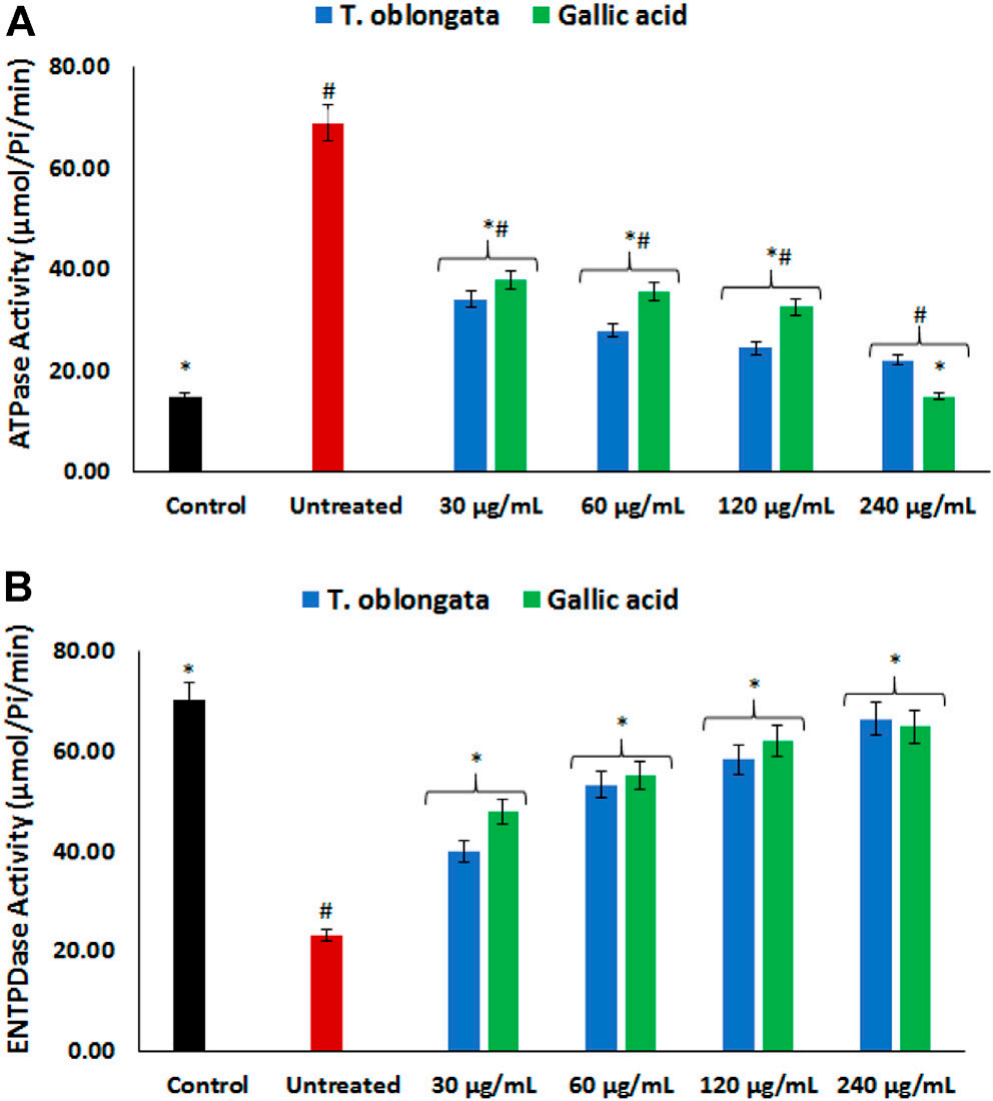
Effect of *T. oblongata* on **(A)** atpase and **(B)** entpdase activities in oxidative cardiotoxicity. Data = mean ± SD; *n* = 3. *Statistically significant compared to untreated cardiac tissues; #statistically significant compared to the control (normal cardiac tissues).

As depicted in Figures 3A,B, there was a significant (*p* < 0.05) elevation in cardiac ATPase activity, with concomitant decreased ENTPDase activity following the induction of oxidative cardiac injury. Treatment with the DCM extract led to significant (*p* < 0.05) reversion of these activities to near normal dose-dependently and compared favorably with gallic acid.

**FIGURE 4:**
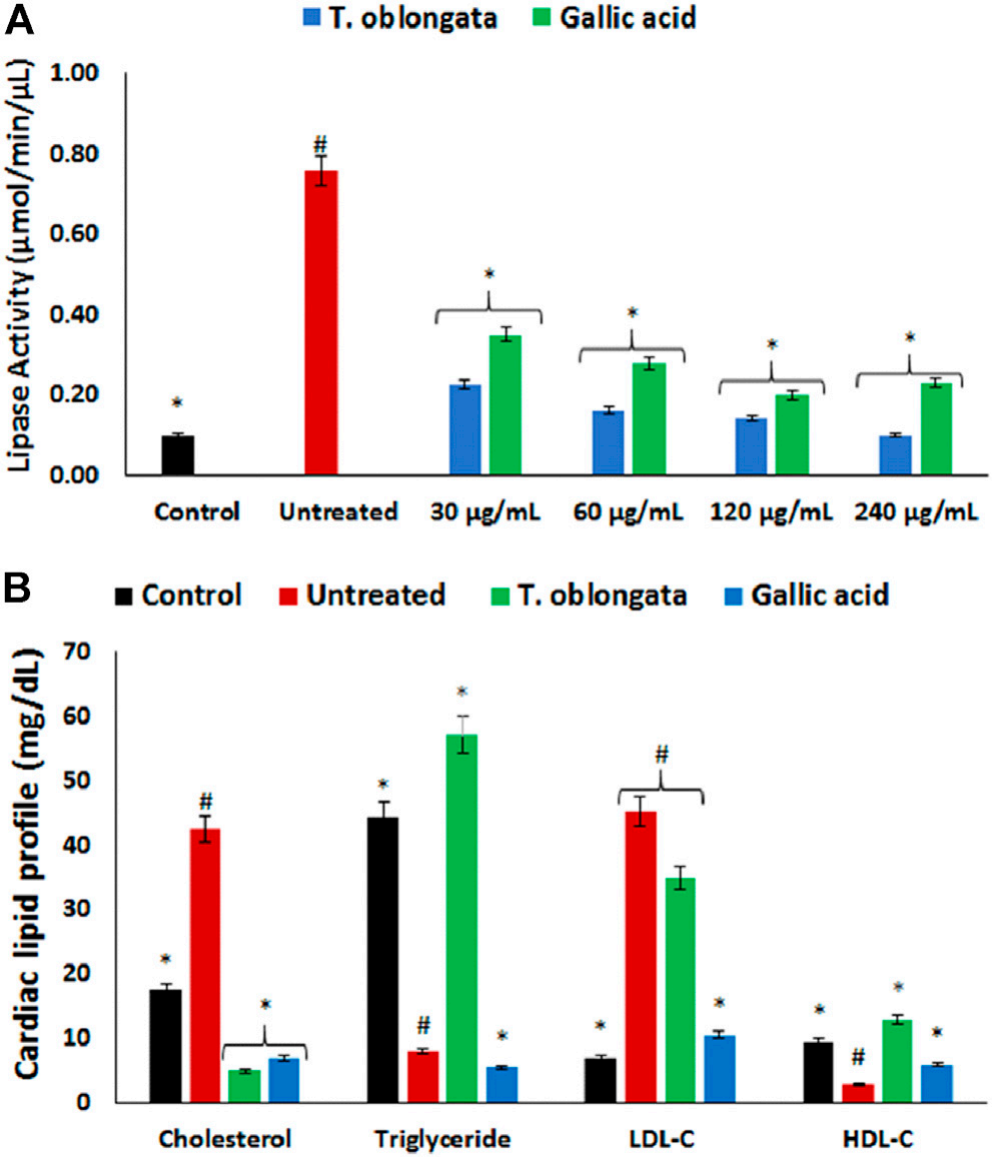
Effect of *T. oblongata* on **(A)** lipase activity and **(B)** lipid profile in oxidative cardiomyopathy. Data = mean ± SD; *n* = 3. *Statistically significant compared to untreated cardiac tissues; #statistically significant compared to the control (normal cardiac tissues).

There was a significant (*p* < 0.05) elevation in cardiac lipase activity following the induction of oxidative cardiac injury as shown in Figure 4A. The activity was significantly (*p* < 0.05) inhibited dose-dependently after treatment with the DCM extract, and compared favorably with gallic acid.

Induction of oxidative cardiac injury led to significant (*p* < 0.05) elevation of cardiac levels of cholesterol and LDL-C, with concomitant decreased levels of triglycerides and HDL-C as shown in Figure 4B. Following treatment with the DCM extract, there was a significant depletion in cardiac level of cholesterol with concomitant elevation of triglyceride and HDL-C levels. Although the level of LDL-C was reduced in the treated tissues, it was however not statistically significant.

GC-MS analysis of the extracted lipid metabolites revealed the presence of unsaturated fatty acid, saturated fatty acid, fatty ester, fatty alcohol, fatty amide, glycerol and steroids in the cardiac tissue as shown in Table 1. Induction of cardiac tissue led to the complete depletion of octadecadienoic acid (Z,Z)-; cis-10-heptadecenoic acid; eicosanoic acid; n-heptadecanol-1 and retinal; with concomitant generation of 14-pentadecenoic acid; octadecanoic acid; tetracosanoic acid; cis-11,14-eicosadienoic acid, methyl ester; n-tetracosanol-1 and glycerol 1-palmitate. Treatment with the DCM extract led to the depletion of oxidative generated 14-pentadecenoic acid; tetracosanoic acid; cis-11,14-eicosadienoic acid, methyl ester; n-tetracosanol-1 and glycerol 1-palmitate, with concomitant generation of 9,12-Octadecadienoic acid (Z,Z)-; cis-11-eicosenoic acid; malonic acid, 2-hexyl tetradecyl ester; l-(+)-Ascorbic acid 2,6-dihexadecanoate; pentanoic acid, heptadecyl ester; 13-tetradecen-1-ol acetate; oleamide and pseduosarsasapogenin-5,20-dien methyl ether. Non lipid metabolites consisting of ursane-3,12-diol; betulin and 28-hydroxylup-20(29)-ene-3,21-dione were also identified in cardiac tissues treated with the DCM extract.

**FIGURE 5:**
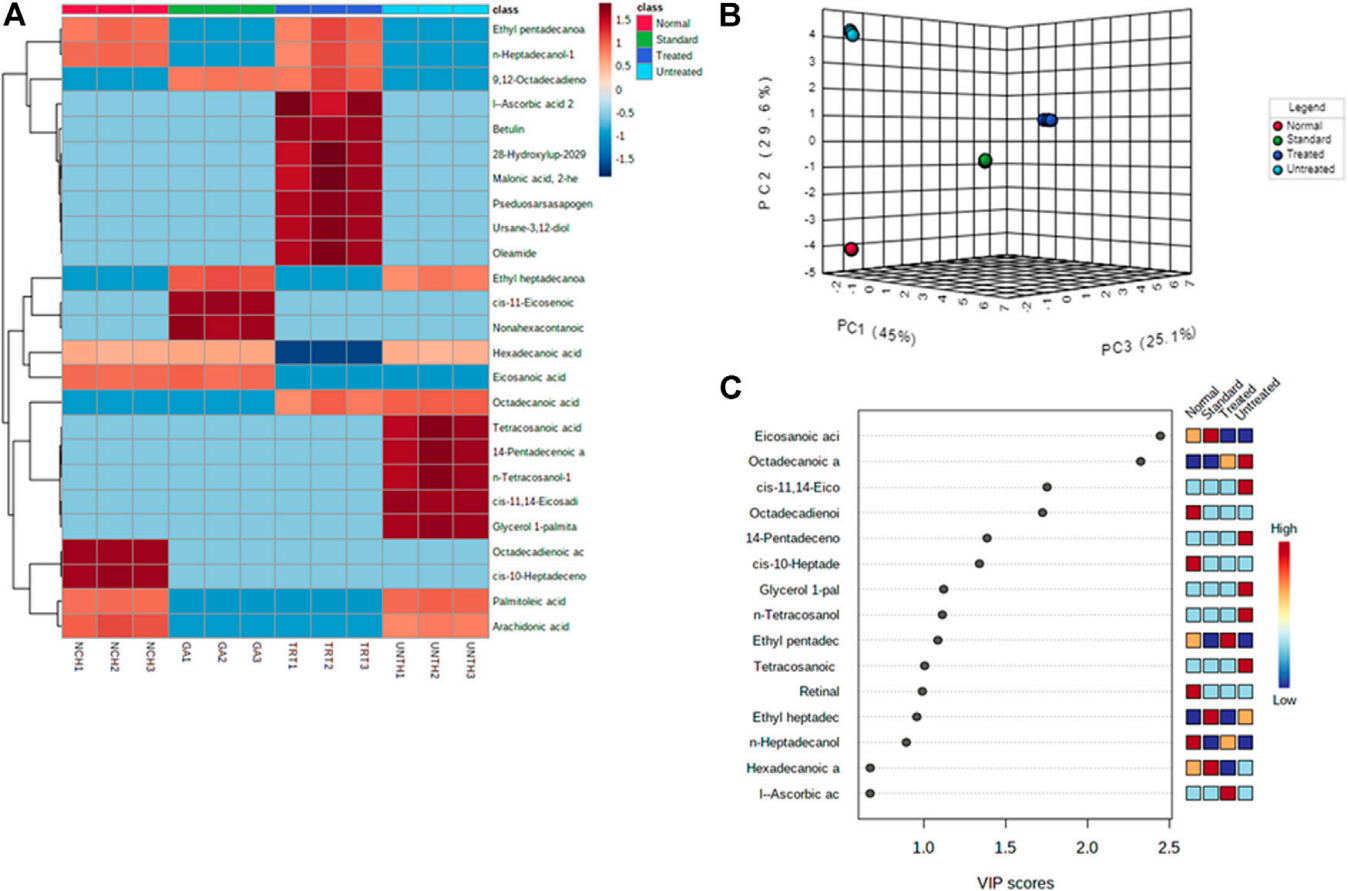
**(A)** Heat map; **(B)** PC and **(C)** VIP scores of identified cardiac lipid metabolites. NCH = normal cardiac tissues; UNTH = untreated cardiac tissues; TRT: T. oblongata–treated cardiac tissues; and GA: gallic acid–treated cardiac tissues.

Distinct changes and distribution of the cardiac metabolites were observed for all the experimental groups as portrayed by the negative values and heat intensity of the heat map following clustering analysis (Figure 5A). This is also supported by the distinctive changes between the pair wise score plots between the selected principal components (PCs) of metabolites from the treated tissues and that of the untreated (Figure 5B). This is further corroborated by the distribution of the important identified features (metabolites) and their respective concentrations in each experimental group by Partial Least Squares - Discriminant Analysis (PLS-DA) as shown in Figure 5C.

**FIGURE 6:**
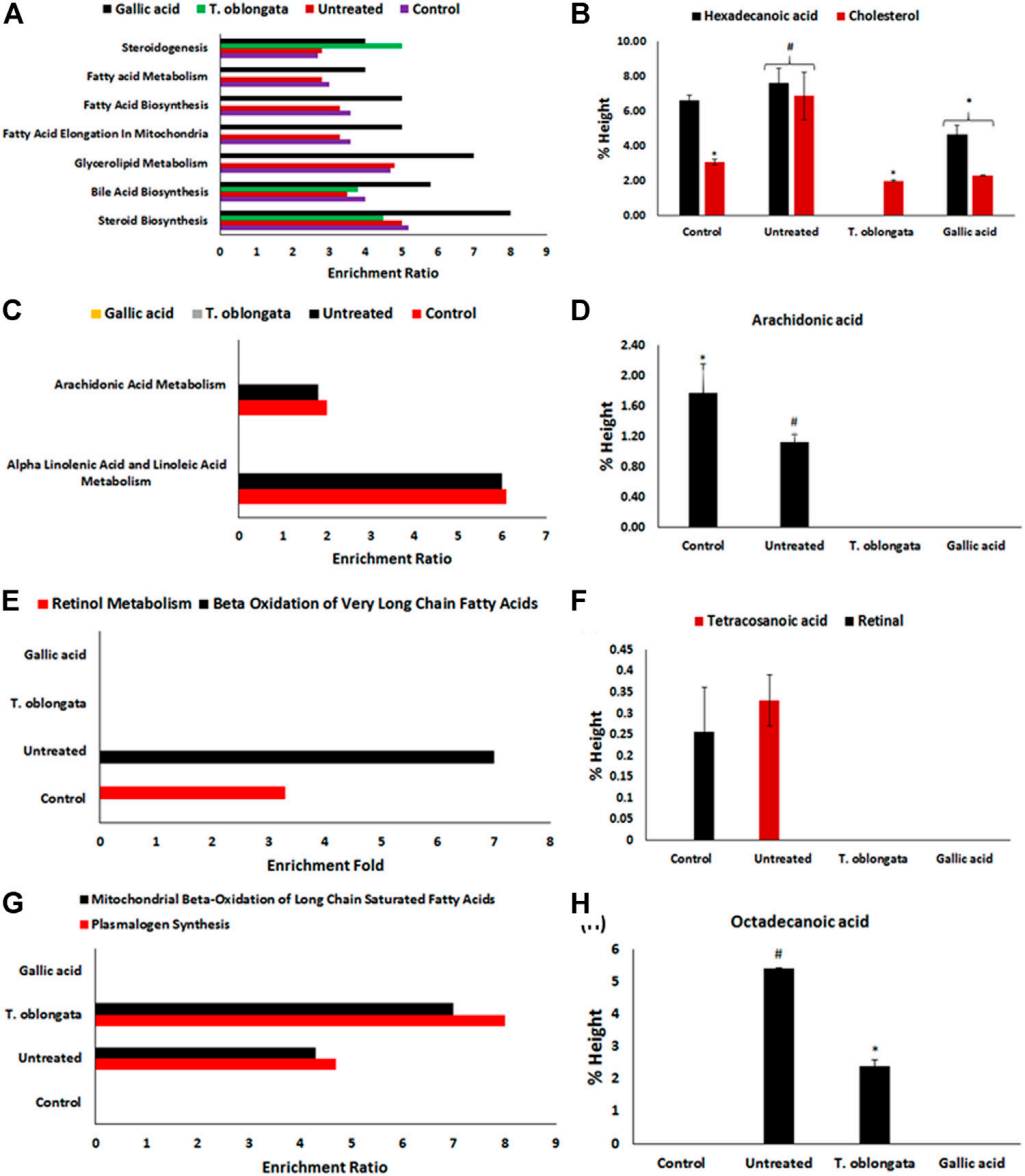
**(A)** Fold enrichment for steroid biosynthesis, bile acid biosynthesis, glycerolipid metabolism, fatty acid elongation in mitochondria, fatty acid biosynthesis, fatty acid metabolism and steroidogenesis pathways and their **(B)** metabolites concentrations; **(C)** fold enrichment for alpha linolenic acid and linoleic acid metabolism, and arachidonic acid metabolism pathways and their **(D)** metabolites; **(E)** fold enrichment for beta oxidation of very long chain fatty acids and retinol metabolism pathways and their **(F)** metabolites; **(G)** fold enrichment for plasmalogen synthesis and mitochondrial beta-oxidation of long chain saturated fatty acids and their **(H)** metabolites.

Pathway enrichment of the identified cardiac lipid metabolites revealed an inactivation of retinol metabolism pathway, with concomitant activation of beta oxidation of very long chain fatty acids, plasmalogen synthesis and mitochondrial beta-oxidation of long chain saturated fatty acids pathways as depicted in Figure 6 and Table 2. Treatment with the DCM extract did not reactivate the retinol metabolism pathway but inactivated oxidative-activated beta oxidation of very long chain fatty acids. It further inactivated arachidonic acid metabolism, glycerolipid metabolism, fatty acid elongation in mitochondria, fatty acid biosynthesis, fatty acid metabolism and steroidogenesis pathways. The oxidative-activated pathways were inactivated in cardiac tissues treated with gallic acid.

**TABLE 2:** Identified pathways in experimental cardiac tissues.

Pathways	Normal	Untreated	*T. Oblongata*	Gallic acid
Steroid biosynthesis	X	X	X	X
Bile acid biosynthesis	X	X	X	X
Alpha linolenic acid and linoleic acid metabolism	X	X	–	–
Glycerolipid metabolism	X	X	–	X
Fatty acid elongation in mitochondria	X	X	–	X
Fatty acid biosynthesis	X	X	–	X
Retinol metabolism	X	–	–	–
Fatty acid metabolism	X	X	–	X
Steroidogenesis	X	X	X	X
Arachidonic acid metabolism	X	X	–	–
Beta oxidation of very long chain fatty acids	–	X	–	–
Plasmalogen synthesis	–	X	X	–
Mitochondrial beta-oxidation of long chain saturated fatty acids	–	X	X	–

X = detected; – = not detected.

**FIGURE 7:**
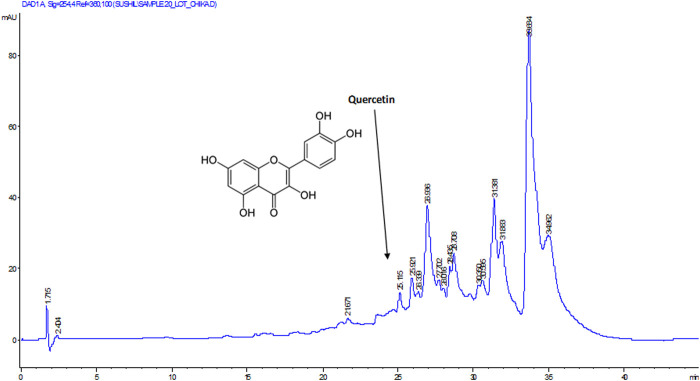
HPLC chromatogram of DCM extract of *T. oblongata*.

To determine the bioactive compound that may be responsible for the studied biological activity of the DCM extract, the extract was subjected to HPLC analysis. As depicted in Figure 7, quercetin was identified as the active compound in the extract.

## Discussion

Cardiotoxicity is a major global health issue as it has been implicated in the pathogenesis of cardiovascular dysfunction which may lead to cardiac failure if not well treated and managed (Poulter, 2003; Wold et al., 2005; Li et al., 2019). Several factors including lipotoxicity, oxidative imbalance, exacerbated ACE activity and altered bioenergetics have been implicated in the pathophysiology of cardiovascular diseases. The high cost associated with its treatment coupled with the side effect of synthesized drugs are of major concerns to health practitioners. This have led to an increased paradigm shift to natural products for cheap and affordable drugs with little or no side effects. The present study reports the cardioprotective effect of *T. oblongata* and the possible mechanism and pathways by which it brings about its effect.

Oxidative stress has been implicated as a major mechanism in the pathogenesis and progression of cardiotoxicity and other cardiological dysfunctions (Fearon and Faux, 2009; Angsutararux et al., 2015). This has been attributed to suppressed endogenous antioxidant activity owing to increased free radical productions in the cardiac tissues. In the present study, the depleted GSH level, SOD and catalase activities (Figures 1A–C) depicts an onset of oxidative stress following induction of oxidative cardiac injury. This is further evidenced by the elevated cardiac MDA level (Figure 1D) which indicates a peroxidative effect on the cardiac lipids following the induction of oxidative cardiac injury. These altered activities and levels correspond with previous reports on the occurrence of oxidative stress after induction of oxidative injury in isolated hearts (Erukainure et al., 2020a; Salau et al., 2020b). The onset of oxidative stress can be attributed to iron-catalyzed Fenton’s and Haber-Weiss reactions following the incubation of heart tissues with Fe^2+^. The exacerbated GSH level, SOD and catalase activities, with concomitant suppressed MDA level in cardiac tissues treated with the DCM extract of *T. oblongata* indicate an antioxidative and anti-peroxidative effect of the extract. This corroborates previous reports on the antioxidant cardioprotective effect of plants (Wattanapitayakul et al., 2005; Adegbola et al., 2017). The antioxidative activity of the extract may be attributed to the identified flavonoid, quercetin (Figure 7). Quercetin is among the common flavonoids found in most fruits, herbs and vegetables, and has been reported for its potent antioxidant and cardioprotective properties (Zhang et al., 2011; Chen et al., 2013).

Increased ACE activity has been implicated in the pathophysiology of cardiotoxicity and other cardiovascular dysfunctions (Ferrario and Strawn, 2006; Salau et al., 2020b). The enzyme catalyzes the hydrolysis of angiotensin I to angiotensin II, with the latter possessing a vasoconstrictive effect leading to hypertension, myocyte death and hypertrophy (Fiordaliso et al., 2000; Agunloye et al., 2019). The increased ACE activity following the induction of oxidative cardiac injury (Figure 2A), therefore suggests an exacerbated cardiac level angiotensin II. The depleted ACE activity following treatment with the DCM extract of *T. oblongata* indicates an ACE-inhibitory effect of the extract, thus depicting reduced cardiac levels of angiotensin II. This corroborates the ability of the extract to inhibit ACE activity *in vitro* (Table 1 and Supplementary Figure S1). ACE-inhibitors have been reported as potent therapies in the treatment and management of cardiovascular diseases, which may be part of the possible mechanism by which the extract protect against oxidative-mediated cardiotoxicity. This corroborates previous reports on the use medicinal plants as ACE inhibitors (Khan and Kumar, 2019; Le et al., 2020). The ACE inhibitory activity of the extract may be attributed to the presence of quercetin, which has also been reported for its ACE inhibitory activity (Huang et al., 2017; Luo et al., 2017).

The elevated acetylcholinesterase activity following the induction of oxidative cardiac injury (Figure 2B) suggests a decreased cardiac level of acetylcholine, thus depicting a cholinergic dysfunction. This can be attributed to the fact the enzyme catalyzes the hydrolysis of acetylcholine to acetate and choline. Acetylcholine is important in muscular contraction and also facilitates vasodilation *via* the muscarinic receptors (Kellogg et al., 2005; Agunloye et al., 2019), thus indicating that decreased concentrations could have detrimental effect on the normal physiological function of the heart. Several studies have reported acetylcholinesterase inhibition as a therapeutic mechanism in the treatment and management of cardiotoxicity and other cardiovascular dysfunctions (Gavioli et al., 2014; Roy et al., 2014; Wu et al., 2017). The decreased acetylcholinesterase in the treated cardiac tissues therefore suggests a therapeutic effect against oxidative-mediated cardiotoxicity. This corresponds with several reports on the ability of medicinal plants to inhibit cardiac acetylcholinesterase activities (Agunloye et al., 2019; Salau et al., 2020b).

Purinergic enzyme activities have been reported to play an important role in the normal physiological activities of the heart. These enzymes phospho-hydrolyze adenosine triphosphate (ATP) and adenosine monophosphate (AMP) to produce the endogenous signaling nucleotide, adenosine which has been reported as a potent dilator and an important factor in bioenergetics (Das and Maulik, 1996; Burnstock, 2017). The increased ATPase and decreased ENTPDase activities following the induction of oxidative cardiac injury (Figures 3A,B) indicate a decreased cardiac level of ATP and adenosine. These levels in the untreated cardiac tissues corresponds with previous reports on altered purinergic activities in oxidative injuries (Salau et al., 2020a; Erukainure et al., 2020b). The increased ATPase activity suggests a diminished cardiac level of ATP and depicts an altered bioenergetic activity as ATP is an important energy-signaling molecule. Altered cardiac bioenergetics have been implicated in the pathophysiology of oxidative stress, cardiotoxicity and other cardiovascular dysfunctions (Das and Maulik, 1996). Both ATP and adenosine have been reported for their vasodilatory activities in coronary vessels (Kirby et al., 2010). The decreased ATPase and elevated ENTPDase activities following treatment with *T. oblongata* DCM extract therefore indicates an improved purinergic activity which may suggest improved bioenergetics.

The normal heart function depends on lipids as its energy fuel for its physiological functions *via* hydrolysis of cardiac triglyceride by lipase to fatty acids (Ritterhoff and Tian, 2017). The heart does not produce triglyceride and thus depends on its supply from the liver. The elevated lipase activity following the induction of oxidative cardiac injury (Figure 4A) therefore suggests an incessant breakdown of cardiac triglyceride, which corroborates with the depleted cardiac level of triglyceride (Figure 4B). The elevated lipase activity and reduced triglyceride level in the untreated cardiac tissues suggests abnormality in FA supply and utilization. This may further indicate a suppressed ATP level which also corroborates the increased ATPase activity (Figure 3A). This may therefore portray a metabolic dependence on aerobic glycolysis for cardiac energy (ATP) supply (Stanley, 2001; Salau et al., 2020b). The depleted lipase activity and elevated triglyceride level in cardiac tissues treated with *T. oblongata* extract therefore indicate an arrest of incessant lipolysis of triglyceride and improved lipid utilization, which also suggests an improved cardiac bioenergetic.

The elevated cholesterol and LDL-C, with concomitant depleted HDL-C following the induction of oxidative cardiac injury (Figure 4B) indicates a disrupted cardiac lipid spectrum and insinuate a cardiac lipotoxic effect which has been linked to the pathophysiology of cardiotoxicity (Poulter, 2003). High concentrations of cholesterol and LDL-C have also been implicated in vasoconstriction and linked to most cardiovascular dysfunctions (Lamping et al., 1994; Pfister and Campbell, 1996; Kopkan et al., 2009). The decreased cholesterol and LDL-C levels as well as elevated HDL-C level on treatment with *T. oblongata* therefore indicates an antilipemic effect of the extract against oxidative-disrupted cardiac lipid profile. This correlates with previous reports on the ability of medicinal plants to maintain cardiac lipid spectrum in oxidative-mediated cardiotoxicity (Erukainure et al., 2020a).

An occurrence of cardiac lipotoxicity on induction of oxidative cardiac injury was further depicted by dysregulated lipid metabolic pathways and altered lipid metabolites (Tables 2, 3 and Figures 5, 6). Dysregulated lipid metabolism in the heart leading to free fatty acids (FFAs) have been implicated among the pathophysiology of cardiotoxicity and other cardiovascular dysfunctions (Antozzi and Zeviani, 1997; Ventura-Clapier et al., 2004). The elevated cardiac level of cholesterol in the untreated tissues corroborates that of the cardiac lipid spectrum (Figure 4A) and the major metabolites for steroid biosynthesis, bile acid biosynthesis and steroidogenesis pathways (Figures 6A,B). The inactivation of retinol metabolism in the untreated cardiac tissue can be attributed to the complete depletion of retinal (Figures 6E,F) and corroborates previous reports linking the downregulation of the pathway to impaired cardiac lipid metabolism and heart failure (Osorio et al., 2002; Lee et al., 2014). The activation of beta oxidation of very long chain fatty acids and mitochondrial beta-oxidation of long chain saturated fatty acids pathways can be attributed to the metabolites octadecanoic acid and tetracosanoic acid (Figures 6E–H). The incessant activation of these pathways has been implicated in increased production of NADH, FADH_2_ and acetyl-CoA (Marín-García and Goldenthal, 2002). Acetyl-CoA is further broken down in the tricarboxylic acid (TCA) cycle to generate NADH, FADH_2_ and ATP. High concentrations of these electron carriers have been reported to cause a high mitochondrial membrane potential leading to the inhibition of the electron transport (Brownlee, 2001; Du et al., 2001). This automatically reduces oxygen (O_2_) to superoxide (O_2_^−^). Thus, it can be insinuated that the continuous production of O_2_^−^ and concomitant low SOD activity (Figure 1B) in the untreated tissues may be an oxidative mechanism on induction of oxidative cardiac injury.

**TABLE 3:** GC-MS identified lipid metabolites in experimental cardiac tissues.

Classes	Compounds	Control	Untreated	*T. oblongata*	Gallic acid
Unsaturated fatty acid	Palmitoleic acid	1.84 ± 0.18	2.62 ± 0.16	ND	ND
	Octadecadienoic acid (Z,Z)-	3.56 ± 0.12	ND	ND	ND
	*cis*-10-Heptadecenoic acid	0.87 ± 0.10	ND	ND	ND
	Eicosanoic acid	5.19 ± 0.10	ND	ND	3.49 ± 0.29
	Arachidonic acid	1.78 ± 0.38	1.12 ± 0.10	ND	ND
	14-Pentadecenoic acid	ND	1.34 ± 0.29	ND	ND
	Octadecanoic acid		5.42 ± 0.01	2.38 ± 0.21	ND
	9,12-Octadecadienoic acid (Z,Z)–	ND	ND	1.48 ± 0.15	0.73 ± 0.11
	cis-11-Eicosenoic acid	ND	ND	ND	1.01 ± 0.10
Saturated fatty acid	Hexadecanoic acid	6.60 ± 0.33	7.60 ± 087	ND	4.68 ± 0.50
	Tetracosanoic acid	ND	0.33 ± 0.06	ND	ND
Fatty ester	Ethyl hexadecanoate	1.28 ± 0.24	1.11 ± 0.21	1.39 ± 0.27	1.68 ± 0.27
	Ethyl pentadecanoate	2.78 ± 0.61	2.44 ± 0.50	2.84 ± 0.57	2.72 ± 0.52
	cis-11,14-Eicosadienoic acid, methyl ester	ND	5.06 ± 0.40	ND	ND
	Ethyl heptadecanoate	ND	ND	ND	ND
	Malonic acid, 2-hexyl tetradecyl ester	ND	ND	0.15 ± 0.01	ND
	l–(+)–Ascorbic acid 2,6-dihexadecanoate	ND	ND	11.96 ± 9.16	ND
	Pentanoic acid, heptadecyl ester	ND	ND	0.17 ± 0.01	ND
Fatty alcohol	Pentadecanol	0.37 ± 0.03	1.59 ± 0.30	0.32 ± 0.02	0.85 ± 0.10
	n-Heptadecanol-1	0.86 ± 0.07	ND	0.72 ± 0.09	ND
	n-Tetracosanol-1	ND	0.49 ± 0.09	ND	ND
	13-Tetradecen-1-ol acetate	ND	ND	0.20 ± 0.03	ND
Fatty amide	Oleamide	ND	ND	0.26 ± 0.03	ND
Glycerol	Glycerol 1-palmitate	ND	0.50 ± 0.03	ND	ND
Steroids	Retinal	0.26 ± 0.11	ND	ND	ND
	Cholesterol	3.08 ± 0.18	6.87 ± 1.36	1.99 ± 0.04	2.29 ± 0.02
	Pseduosarsasapogenin-5,20-dien methyl ether	ND	ND	0.34 ± 0.06	
Non-lipid	Ursane-3,12-diol	ND	ND	0.35 ± 0.04	ND
	Betulin	ND	ND	0.38 ± 0.13	ND
	28-Hydroxylup-20(29)-ene-3,21-dione	ND	ND	0.40 ± 0.01	ND
	Nonahexacontanoic acid	ND	ND	ND	0.32 ± 0.02

Values = mean ± SD; *n* = 3. ND = not detected.

Inactivation of beta oxidation of very long chain fatty acids in tissues treated with *T. oblongata* DCM extract therefore suggests a decreased production of the electron carriers which depicts a suppressed production of O_2_^−^. This corroborates the absence of tetracosanoic acid and the high SOD activity. The inactivation of alpha linolenic acid and linoleic acid metabolism, glycerolipid metabolism, fatty acid elongation in mitochondria, fatty acid biosynthesis, and fatty acid metabolism suggests a possible hypolipidemic mechanism by which the extract mitigates oxidative-mediated lipid dysmetabolism in oxidative-mediated cardiotoxicity.

As depicted in Figure 7, quercetin was identified as an active component of the DCM fraction of *T. oblongata*. Quercetin ranks among the most popular phenolics as it is commonly found in fruits, vegetables, and herbs (Zhang et al., 2011). Its potent antioxidant properties have been reported and has been exploited in the treatment and management of various oxidative-mediated ailments such as diabetes, cardiovascular diseases, and cancer (Zhang et al., 2011; Georgiev et al., 2014; Patel et al., 2018). The studied activities of *T. oblongata* in the present study may therefore be attributed to the presence of quercetin, which may also work in synergy with other unidentified phytochemicals.

## Conclusion

These results indicate the cardioprotective effect of *T. oblongata* against oxidative-mediated cardiotoxicity. This is evidenced by the ability of its DCM extract to mitigate lipotoxicity and modulate dysregulated cardiometabolic activities as shown by its antioxidative activity and suppressive effects on ACE, acetylcholinesterase and lipase activities, while modulating cardiac lipid dysmetabolism. The presence of quercetin in the extract may contribute to these activities. However, further *in vivo* studies are recommended to identify the molecular mechanism that maybe involved in the cardioprotective effect of the extract and its bioactive compounds.

## Data Availability

The original contributions presented in the study are included in the article/Supplementary Material, further inquiries can be directed to the corresponding author.
