# Supplementary material for: Turbina oblongata Protects Against Oxidative Cardiotoxicity by Suppressing Lipid Dysmetabolism and Modulating Cardiometabolic Activities Linked to Cardiac Dysfunctions
Source: Front Pharmacol. 2021 May 20;12:610835. doi: 10.3389/fphar.2021.610835 (PMC8174711; doi:10.3389/fphar.2021.610835)
Supplement: Supplementary file 1 [file DataSheet1.PDF]

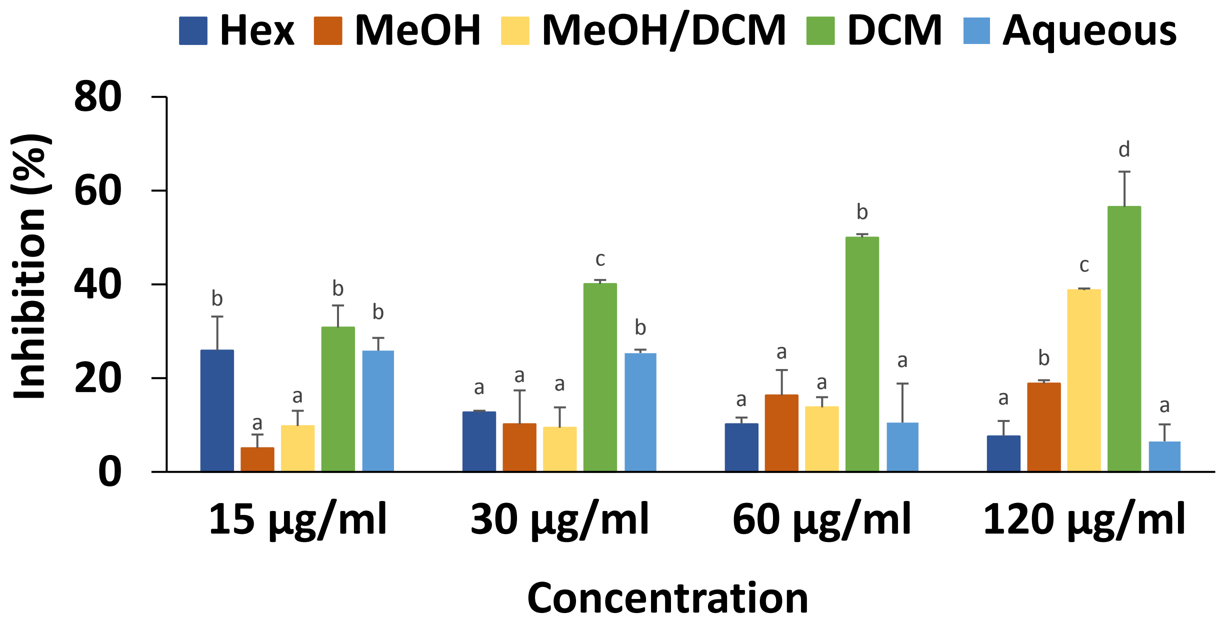

Fig. S1A: ACE inhibitory activity of *T. oblongata* extracts. Values = mean ± SD; n = 3. Alphabets above bars are statistically significant to each other

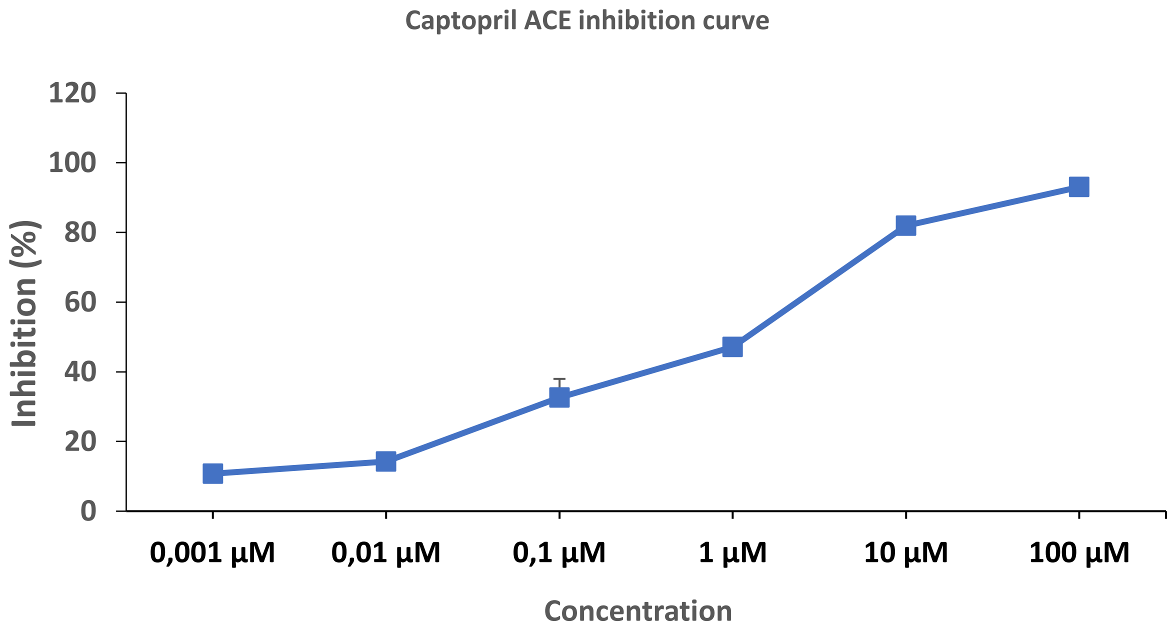

Fig. S1B: ACE inhibitory activity of captopril oil. Values = mean ± SD; n = 3.

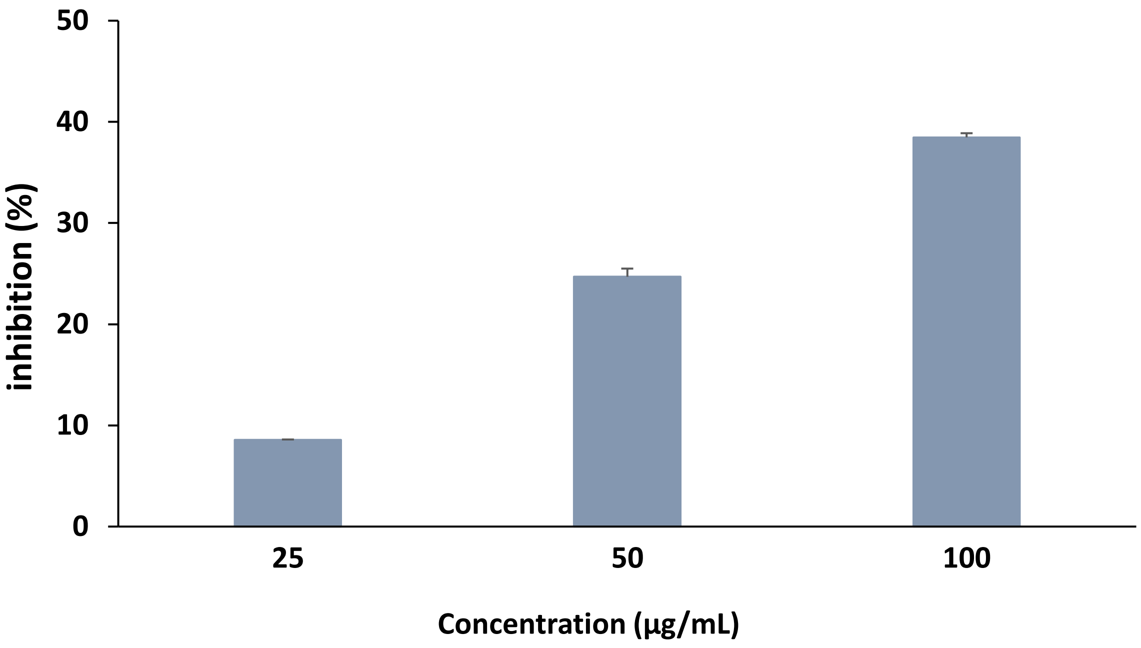

Fig. S2A: Renin inhibitory activity of DCM extract of *T. oblongata*. Values = mean ± SD; n = 3.

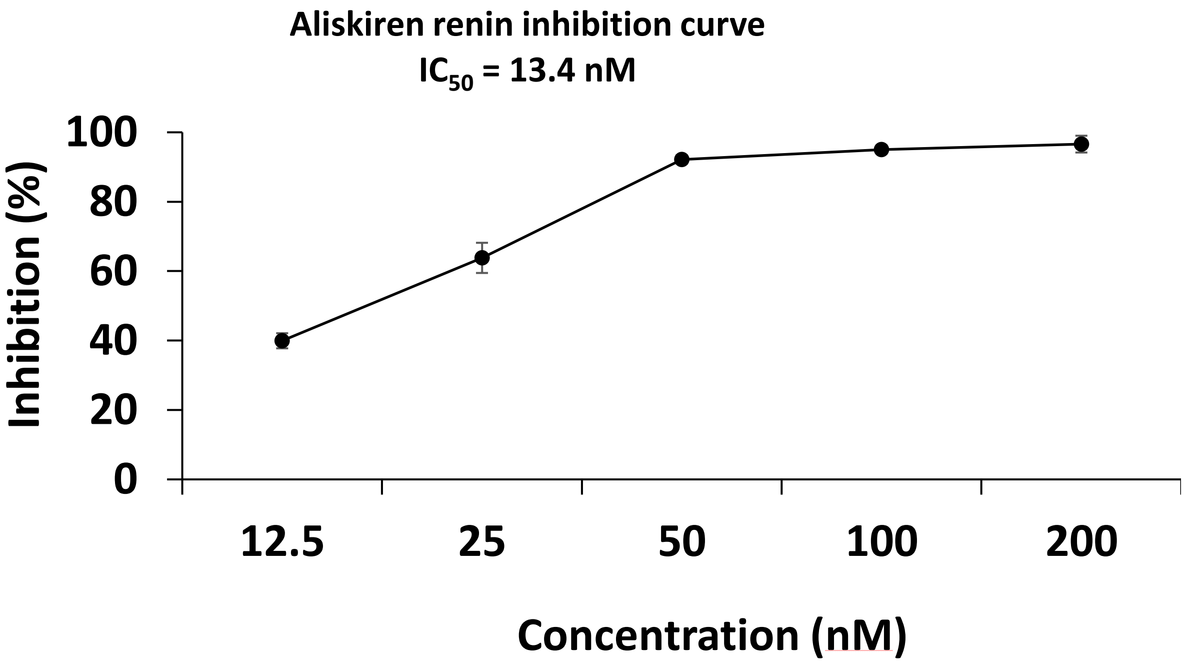

Fig. S2B: Renin inhibitory activity of Aliskiren
